# Sodium–glucose cotransporter 2 inhibitors and the cancer patient: from diabetes to cardioprotection and beyond

**DOI:** 10.1007/s00395-024-01059-9

**Published:** 2024-06-27

**Authors:** Massimiliano Camilli, Marcello Viscovo, Luca Maggio, Alice Bonanni, Ilaria Torre, Claudio Pellegrino, Priscilla Lamendola, Lorenzo Tinti, Luciana Teofili, Stefan Hohaus, Gaetano Antonio Lanza, Peter Ferdinandy, Zoltan Varga, Filippo Crea, Antonella Lombardo, Giorgio Minotti

**Affiliations:** 1 Department of Cardiovascular and Pulmonary Sciences, Catholic University of the Sacred Heart, Rome, Italy; 2 Department of Cardiovascular Medicine, Fondazione Policlinico Universitario A. Gemelli IRCCS, L.go A. Gemelli, 1, 00168 Rome, Italy; 3 Sezione di Ematologia, Dipartimento di Scienze Radiologiche ed Ematologiche, Università Cattolica del Sacro Cuore, Rome, Italy; 4 Dipartimento di Diagnostica per Immagini, Radioterapia Oncologica ed Ematologia, Fondazione Policlinico Universitario A. Gemelli IRCCS, Rome, Italy; 5 Department of Pharmacology and Pharmacotherapy, Semmelweis University, Budapest, Hungary; 6 Pharmahungary Group, Szeged, Hungary; 7 MTA-SE System Pharmacology Research Group, Department of Pharmacology and Pharmacotherapy, Semmelweis University, Budapest, Hungary; 8 HCEMM-SU Cardiometabolic Immunology Research Group, Budapest, Hungary; 9 MTA-SE Momentum Cardio-Oncology and Cardioimmunology Research Group, Budapest, Hungary; 10 Center of Excellence of Cardiovascular Sciences, Ospedale Isola Tiberina - Gemelli Isola, Rome, Italy; 11 University and Fondazione Campus Bio-Medico, Rome, Italy

**Keywords:** Sodium–glucose cotransporter 2 inhibitors, Cardio-oncology, Cardioprotection, Heart failure, Cancer patients

## Abstract

Sodium–glucose cotransporter 2 inhibitors (SGLT2i), a new drug class initially designed and approved for treatment of diabetes mellitus, have been shown to exert pleiotropic metabolic and direct cardioprotective and nephroprotective effects that extend beyond their glucose-lowering action. These properties prompted their use in two frequently intertwined conditions, heart failure and chronic kidney disease. Their unique mechanism of action makes SGLT2i an attractive option also to lower the rate of cardiac events and improve overall survival of oncological patients with preexisting cardiovascular risk and/or candidate to receive cardiotoxic therapies. This review will cover biological foundations and clinical evidence for SGLT2i modulating myocardial function and metabolism, with a focus on their possible use as cardioprotective agents in the cardio-oncology settings. Furthermore, we will explore recently emerged SGLT2i effects on hematopoiesis and immune system, carrying the potential of attenuating tumor growth and chemotherapy-induced cytopenias.

**Supplementary Information:**

The online version contains supplementary material available at 10.1007/s00395-024-01059-9.

## Introduction

Sodium‐glucose cotransporter 2 inhibitors (SGLT2i) represent a class of antidiabetic medications with a unique story. Initially approved for the treatment of type 2 diabetes mellitus, thanks to their glycosuric action, subsequently showed cardioprotective and nephroprotective effects that made them one of the most innovative drugs in cardiology and nephrology over the last decades [20, 164].

SGLT2i reduce serum glucose by inhibiting renal tubular glucose reabsorption, thus promoting urinary glucose excretion [143]. Two types of sodium‐glucose cotransporters are present in the nephron, mainly located in the S2 and S3 segments of the proximal tubule, reabsorbing the majority of filtrated urinary glucose [155]. By inhibiting SGLT2, these drugs impair glucose reabsorption in the proximal tubule, determining a reduction of the renal threshold of glycosuria. Canagliflozin, dapagliflozin and empagliflozin are representatives of SGLT2i, also known as gliflozins [97].

An important hemodynamic effect of gliflozins is related to blood pressure reduction, through tubulo-glomerular feedback stimulation reversal, glycosuria and natriuresis. Moreover, SGLT2i may improve blood pressure control by inhibiting the sympathetic nervous system [100]. Of importance, SGLT2i promote weight loss and lipid metabolism shift, inducing visceral fat reduction, as well as increased lipolysis [72, 133]. The ramifications of SGLT2 inhibition in lipid metabolism also enable to understand their atheroprotective effect [8, 133]. In this context, an emerging property of gliflozins is related to their anti-inflammatory action, causing the attenuation of interleukin (IL)-6, tumor necrosis factor (TNF), interferon (IFN)-γ, NF-κβ, toll-like receptor (TLR)-4 and transforming growth factor (TGF)-β [17, 74]. Both in vitro and in vivo models showed that gliflozins may limit inflammation, targeting a wide array of pathways that play a pivotal role in the development of atherosclerosis [107, 128].

In 2015, the EMPA-REG OUTCOME trial was the first study to focus on the cardiovascular outcomes related to treatment with SGLT2i. In 7020 patients with type 2 diabetes mellitus and high cardiovascular risk, randomized to empagliflozin or placebo and evaluated at a median follow-up of 3.1 years, a reduction in heart failure (HF) hospitalization, cardiovascular death and mortality was observed in the empagliflozin arm compared to placebo [13]. These promising results laid the groundwork for successful trials with SGLT2i also in HF patients with either reduced or preserved ejection fraction (HFrEF and HFpEF, respectively) [6, 91, 130], afterwards confirmed by several meta-analyses [19, 162]. Overall, available evidences made SGLT2i one of the “four pillars” of HFrEF treatment and the first recommended medication in HF with mildly-reduced ejection fraction (HFmrEF) and HFpEF, according to most recent updates of guidelines [89, 90].

Growing data also introduced the possibility of adding SGLT2i to the pharmacological armamentarium of cardiovascular disease prevention [33] or treatment of subclinical cardiac dysfunction, including those cases observed in cancer patients exposed to potentially-cardiotoxic agents [21, 95]. At the same time, preclinical data suggested a role of SGLT2i in preventing cancer progression and mitigating cytopenia induced by chemotherapy [95].

**Fig. 1:**
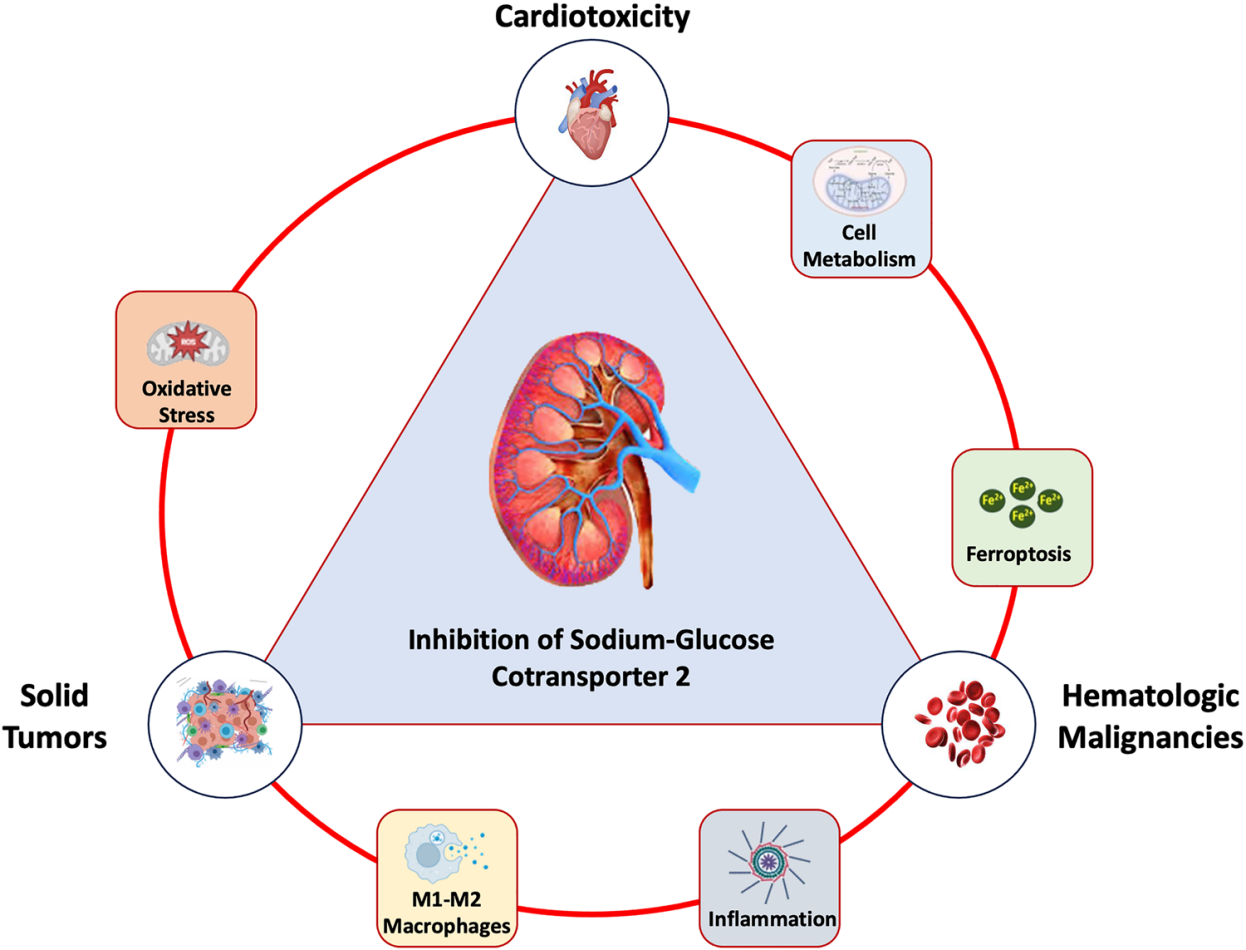
Role of sodium glucose cotransporter 2 (SGLT2) inhibitors across the spectrum of cardiotoxicity, solid tumors and hematologic malignancies

This review will, therefore, focus on the role of SGLT2i as modulators of the cardiovascular system, with special emphasis being paid to myocardial function and metabolism pathways. The biological rationale and clinical evidence for bringing SGLT2i at the center of the Cardio-Oncology stage will be presented (Fig. 1). We will also shed light on possible research directions and their implications in cancer patients’ management.

## Mechanisms of cardiac protection in cancer patients through SGLT2 inhibition

In preclinical models, SGLT2i have consistently shown to exert direct beneficial effects on the cardiac tissue, especially in the setting of chronic conditions such as HF. These effects reflect SGLT2i modulation of pro-inflammatory and oxidative stress processes, ion transport, myocardial sodium and calcium homeostasis, as well as metabolic/mitochondrial pathways, such as ketone bodies production [25, 96, 120, 164]. Given that these processes are also supposed to be involved in chemotherapy-associated cardiac injury, there is growing interest in the potential role played by SGLT2i in the field of Cardio-Oncology [31].

### Biological basis of SGLT2i protection against anthracycline-induced cardiotoxicity

**Fig. 2:**
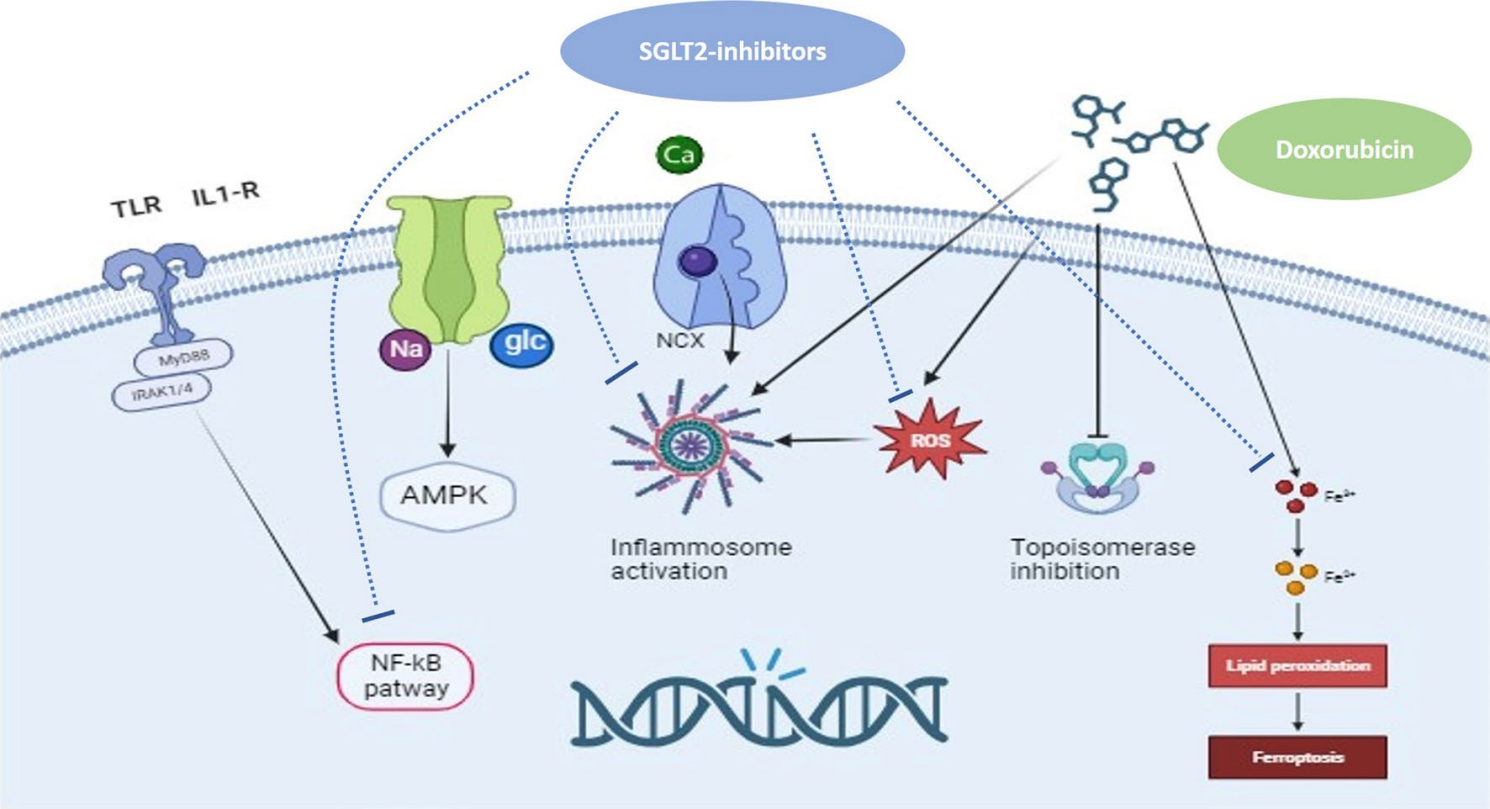
Molecular mechanisms of anthracycline-induced cardiomyocyte death. Anthracyclines, in particular doxorubicin, treatment initiates multiple pathways including upregulation of death receptors, calcium overload, disruption in iron homeostasis with lipid peroxidation, generation of ROS with final DNA damage and cell death. *AMPK* 5ʹ adenosine monophosphate-activated protein kinase, *DNA* deoxyribonucleic acid, *Fe2+* iron, *GLC* glucose, *MyD88* Myeloid differentiation primary response 88, *IL-1β R* Interleukin-1 beta receptor, *NF-kB* Nuclear factor kappa-light-chain-enhancer of activated B cells, *ROS* reactive oxygen species, *TLR* toll-like receptor

Anthracyclines represent the backbone treatment for several solid and hematological malignancies and are associated with potential dose-dependent cardiotoxic effects, eventually leading to the development of subclinical left ventricular dysfunction, overt HF and malignant arrhythmias [125]. There is a plethora of mechanisms involved in anthracycline-induced cardiotoxicity, among others (i) regulation of macrophage phenotype expression; (ii) generation of highly reactive oxygen species (ROS) inducing membrane lipid peroxidation and causing a direct damage to cardiac myocytes [52–54]; (iii) inhibition of cardiac topoisomerase IIβ, leading to transcriptome changes, mitochondrial dysfunction and apoptosis [56, 116, 126, 134, 135, 137, 159]; (iv) downregulation of ferroptosis. Main mechanisms of anthracycline induced cardiotoxicity are sketched in Fig. 2.

#### Regulation of macrophage phenotype expression

Macrophages are important actors of immune response, being involved in both activation and resolution of the inflammatory process, and in tissue repair through fibrosis [64]. In brief, the different roles played by macrophages are explained by their phenotype expression, with M1 and M2 macrophages exerting, respectively, induction and resolution of inflammation [85].

M2 macrophages have been shown to influence post-myocardial infarction remodeling in mice models through the action of IL-13, which modulates monocyte infiltration in the wounded cardiac tissue and their polarization [55]. M2 macrophages contribute to the control of the inflammatory process through several actions; in particular, M2a macrophages are associated with arginase production, which is believed to determine collagen deposition and fibrosis development. On the other hand, M2c macrophages, preferentially activated by SGLT2i, are believed to favor IL-10 expression, a suppressor cytokine that promotes the resolution of the fibrotic process [76]. It was in keeping with these notions that four weeks treatment with SGLT2i promoted cardiac repair and reduced pathological remodeling in a rat model of myocardial infarction [76].

SGLT2i also attenuated cardiac fibrosis via regulating the signal transducer and transcription activator STAT3-dependent pathway, consequently reducing infiltration of myofibroblast and collagen accumulation [76]. At the same time, empagliflozin limited cardiac fibrosis by inhibiting the NADPH-oxidase activity following ischemic injury [79].

Although multiple studies investigated the effects of SGLT2i on macrophages and fibrosis, our understanding of the precise role of cardiac macrophages in doxorubicin-induced cardiotoxicity is still limited. The imbalance between macrophage phenotypes and their infiltration in the myocardium seems to be involved also in anthracycline-induced myocardial injury [39]. Studies in mouse models suggest that acute doxorubicin treatment increases M1 macrophage population, while suppressing the M2 counterpart [15, 73]. Moreover, studies of an IL-12p35-KO mouse mode showed that IL-12 and IL-35 deficiency exacerbated doxorubicin-induced myocardial injury through promotion of M1 macrophage differentiation, increase of pro-inflammatory cytokines and reduction of M2 macrophage-related anti-inflammatory cytokines [157]. Other studies demonstrated that IL-22 is a critical regulator of macrophage differentiation in response to cardiac injury [158], while IL-22 deficiency reverses doxorubicin-induced cardiac imbalance of M1 versus M2 macrophages, increasing the M2 population. This effect was associated with reduced cardiomyocyte vacuolization and apoptosis, with concomitant improvement of global cardiac function [158].

#### *Modulation of cardiomyocyte Ca*^*2*+^*/Na*^+^*homeostasis and downregulation of inflammasome*

The inflammasome is a multiprotein complex that plays a role in the activation and perpetuation of the inflammatory process, with nucleotide-binding oligomerization domain (NOD)-like receptor pyrin domain containing protein 3 (NLRP3) being a critical component of the innate immune system. This mediates caspase-1 activation and the secretion of proinflammatory cytokines in response to microbial infection and cellular damage [61]. The inflammasome, in order to exert its function, needs to be activated through three stimulus events: ionic flux, ROS and/or organelle damage.

Indeed, multiple studies investigated the role of the inflammasome in myocardial injury and its interaction with gliflozins. In acute myocardial infarction, the activation of NLRP3 inflammasome is critical, as shown by the reduced extension of infarct size in Nlrp3−/− mice [61, 93], although its role in the ischemia–reperfusion (I/R) damage is still controversial [58, 123]. In mice models of HF, empagliflozin blunts the decline in cardiac function, also reducing NLRP3 inflammasome activation and IL1β secretion [66]. Likewise, empagliflozin attenuates macrophagic NLRP3 inflammasome activation in patients with type 2 diabetes [68]. As well, a series of reports showed that empagliflozin positively regulates cardiomyocyte homeostasis and inflammasome activity. Of note, in preclinical studies, SGLT2i inhibited the Na^+^/H^+^ exchanger, leading to lower cytosolic concentration of Na^+^ and Ca^2+^ in cardiomyocytes [14, 141]. In diabetic rats, empagliflozin exerts a potential anti-diabetic effect through prevention of Ca^2+^/Na^+^ dysregulation in cardiomyocytes and decrease in ROS, leading to improved cardiac function, attenuation of ventricular hypertrophy and correction of prolonged QT interval [75].

In the Cardio-Oncology field, anthracycline exposure increases levels of ROS and Ca^2+^ in cardiomyocytes, with subsequent mitochondrial dysfunction, inflammasome activation, apoptosis and necrosis [79]. Doxorubicin exposure increases the production of circulating IL-1-β, IL-6 and C reactive-protein (CRP), enhancing through inflammation, the risk of metabolic diseases and cardiovascular manifestations [114]. Indeed, gliflozins counterbalance the various cardiotoxic mechanisms generated by anthracyclines, thus leading to an improvement in dysregulated pathways. Quagliariello et al. demonstrated that incubation of cardiomyocytes from non-diabetic mice with empagliflozin significantly reduced the intracellular Ca^2+^ content and expression of several pro-inflammatory cytokines. Moreover, the authors demonstrated an improvement in LVEF and radial/longitudinal strain after anthracycline exposure in the empagliflozin mice group [66]. This report also highlighted a role of SGLT2i in the modulation of NLRP3 and myeloid differentiation primary response 88 (MyD88)-related pathways, known to be involved in the cytokine release that characterizes HF [111]. Similarly, the role of empagliflozin in preventing doxorubicin-induced myocardial dysfunction in non-diabetic mice was reported by Sabatino et al. In particular, empagliflozin caused 50% less myocardial fibrosis independently of glycaemic control [121].

#### Normalization of SGK1 and ENaC myocardial expression

Serum- and Glucocorticoid-Regulated Kinase 1 (SGK1) is highly expressed in the human and murine heart and is upregulated in many pathophysiological settings, including obesity, heart disease and diabetes. SGK1 regulates the expression of a number of ion channels, including epithelial sodium channel (ENaC), which is upregulated in obesity and diabetes [47]. In insulin-resistant female diabetic mice, increased myocardial expression of SGK1 and ENaC caused myocardial fibrosis and eccentric LV hypertrophy, while empagliflozin reduced SGK1 and ENaC levels, as well as related pro-fibrotic signals, leading to an improved diastolic relaxation. These results suggest that the SGLT2i mediated improvement in cardiac function may be modulated, in part, through reduction in SGK1/ENaC activity [47]. In a mouse model, Zhang et al. also demonstrated that the downregulation of SGK1 pathway reduced doxorubicin-mediated cardiotoxicity through the phosphorylation and nuclear translocation of NFκB, leading to reduction of cardiomyocyte inflammation and apoptosis [166]. This would further identify SGK1 downregulation as a possible mechanism of cardiac protection induced by SGLT2i.

#### Upregulation of ketone body release

Recent in vitro studies suggest that β-hydroxybutyrate (β-OHB), a ketone body that increases during SGLT2i treatment, may protect against anthracyclines by reducing ROS levels and by improving mitochondrial function in cardiomyocytes [102]. On the other hand, ketone serves as an effective source of energy in the failing heart and can mitigate cardiomyocyte damage by reducing ROS and by increasing ATP levels [10]. Modulation of cardiac metabolism might thus represent a rather pivotal mechanism through which SGLT2i could protect against anthracycline cardiotoxicity.

#### Downregulation of ferroptosis

Ferroptosis is a mechanism of iron-dependent non-apoptotic cell death characterized by iron overload and lipid peroxidation. Ferroptosis occurs in many pathophysiologic conditions and may contribute to acute kidney injury, neuronal death, and cancer cell death as well [36, 62, 81, 82, 154]. Ferroptosis is also involved in cardiovascular diseases such as cardiomyopathies and acute myocardial infarction [32]. In a mouse model of cardiac ischemia–reperfusion injury, the iron chelator desferrioxamine and the glutaminolysis inhibitor compound 968, both with ferroptosis inhibitory activity, reduced myocardial infarct size and improved cardiac function [44, 106].

Canagliflozin mitigates ferroptosis, and by doing so it was shown to improve cardiac function in a preclinical model of HFpEF [83]. This raises interest on SGLT2i as potential cardiac protectant against anthracyclines, especially since ferroptosis is strongly involved in anthracycline cardiotoxicity [167]; however, we are unaware of studies that addressed this hypothesis in detail.

#### Upregulation of PGC-1α and mitochondrial protection

In preclinical studies, anthracycline-dependent cardiomyocyte damage was fingerprinted by reduced AMP-activated protein kinase (AMPK) expression and double-strand deoxyribonucleic acid (DNA) breaks, which is consistent with anthracyclines intercalating in DNA and blocking the activity of topoisomerase IIβ that is constitutively expressed in cardiomyocytes [76]. As anticipated earlier, topoisomerase IIβ inhibition triggers transcriptome changes that culminate in mitochondrial dysfunction and apoptosis [145]. Doxorubicin would nonetheless induce anthracycline cardiotoxicity by many other mechanisms, which may range from excess of ROS production in mitochondria to suppression of peroxisome proliferator-activated receptor gamma coactivator-1 alpha (PGC-1α), a transcription coactivator that plays a primary role in mitochondrial oxidative phosphorylation and fatty acid oxidation [117, 146, 160]. Studies of ischemic cardiomyopathy in laboratory animals have shown that empagliflozin increases the levels of PGC-1α, leading to mitochondrial protection [161], while other studies showed that similar mechanisms enabled empagliflozin to protect cardiomyocytes against doxorubicin [144].

**Fig. 3:**
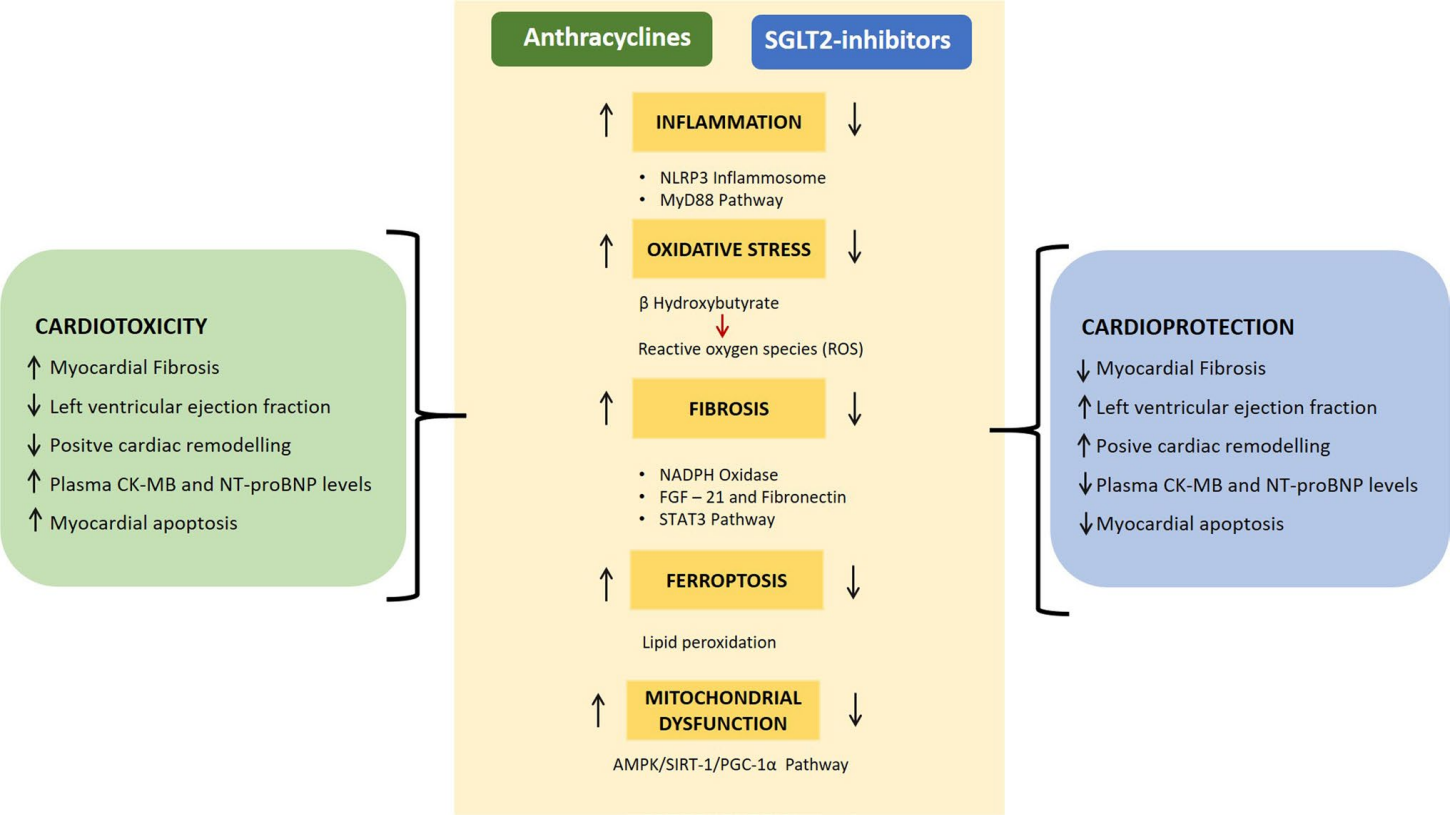
Molecular mechanisms through which SGLT2 inhibitors may exert a cardioprotective role in anthracycline-treated patients and in patients developing cancer therapy related cardiac dysfunction. *AMPK* 5ʹ adenosine monophosphate-activated protein kinase, *CKMB* Creatine Kinase MB, *FGF-21* Fibroblast growth factor-21, *NLRP3* nucleotide-binding oligomerization domain-like receptor family pyrin domain containing 3, *NTproBNP* N-terminal pro B-type natriuretic peptide, *MyD88* Myeloid differentiation primary response 88, *IL-1β* Interleukin-1 beta, *NADPH* nicotinamide adenine dinucleotide phosphate, *IL-10* Interleukin-10, *PGC-1* Peroxisome proliferator-activated receptor-gamma coactivator, *SIRT-1* Sirtuin-1, *STAT-3* Signal transducer and activator of transcription 3

The molecular mechanisms through which SGLT2i might exert cardioprotection against anthracyclines are summarized in Fig. 3.

### Biological basis of cardioprotection in cardiotoxicity induced by other cancer drugs

Cardiotoxicity complicates the clinical use of many anticancer drugs other than anthracyclines [52, 138]. There are few preclinical studies exploring the cardioprotective role of SGLT2i against these drugs.

Trastuzumab is an anti-Human Epidermal Growth Factor Receptor 2 (HER2) antibody, primarily used in the treatment of HER2 overexpressing breast cancers (approximately 15–20% of all diagnoses). Anti-HER2 agents exert cardiotoxicity by a variety of mechanisms that include, among others, DNA-damage and ferroptosis [80, 131]. In vitro and in vivo studies showed that empagliflozin mitigates DNA damage and ferroptosis induced by trastuzumab [94]. In other preclinical studies, empagliflozin was able to ameliorate cardiomyocyte autophagy induced by sunitinib, an angiogenesis inhibitor. This reflect empagliflozin interactions with the AMP-activated Protein Kinase-Mammalian Target of Rapamycin (AMPK-mTOR) signaling pathway but other mechanisms of protection might well involve a reversal of sunitinib-induced microvascular injury and the consequent increase of coronary flow [63, 115].

Interesting data emerging from murine models and clinical studies suggest the potential protective effect of SGLT2i in mitigating cardiovascular toxicity caused by anti-angiogenic therapies. Luseogliflozin showed to upregulate the expression of vascular endothelial growth factor (VEGF)-α in the kidney after ischemia–reperfusion injury in non-diabetic mice [165]; at the same time, Nikolaou et al. demonstrated the protective role of chronic administration of empagliflozin in the myocardial ischemia–reperfusion injury in diabetic mice, sustained by the upregulation of VEGF pathway [101].

Antimetabolite drugs, such as 5-Fluorouracil (5-FU) and Capecitabine, are also associated with serious cardiovascular toxicities, such as coronary spasm, myocardial infarction, arrhythmias and HF [129]. Evidence regarding antimetabolites cardiotoxicity may reveal a cardioprotective role exerted by SGLT2i. In a preclinical study, the administration of empagliflozin in mice treated with 5-FU ameliorated the cardiotoxic effects by different mechanisms including vasodilating effect, antioxidant and anti-inflammatory properties and the downregulation of tumor necrosis factor alpha (TNFα)/ toll like receptors (TLR)/ nuclear factor-κB (NF-κB) pathway [113].

Another example of how SGLT2i can counteract or prevent cardiovascular toxicity from non-anthracycline drugs involves studies on carfilzomib (CFZ), a proteasome inhibitor, currently approved for relapsed/refractory multiple myeloma. Clinical employment of CFZ is limited by cardiovascular toxicity, mainly due to endothelial dysfunction. Canagliflozin abrogated the apoptotic effects of CFZ in cultured endothelial cells, and again this occurred by an AMPK-dependent mechanism [26].

A detailed analysis should be reserved to immune checkpoints inhibitors (ICIs), currently approved for many solid tumors treatment [4]. Main compounds used in clinical practice are reported in Table 1 [4, 9, 35, 49, 59, 110, 150, 168]. ICIs, inducing a non-specific autoimmune response by activating T cells, may lead to immune-related adverse events (irAEs) occurrence [109]. It has been observed that around 60–80% of patients who undergo ICIs treatment may experience irAEs, with 20% of them suffering from high-grade irAEs [65]. As well, among cancer patients who received a combination scheme (e.g. ipilimumab + nivolumab), more than 90% had at least one irAE, and nearly half of them had severe irAEs [67]. These side effects obviously do not spare the heart. However, ICIs-induced cardiotoxicity is a relatively rare complication and frequently manifests with myocarditis. Recent studies have found that SGLT2i may offer a promising approach to reduce all-cause mortality in cancer patients receiving ICIs therapy. Perelman et al. retrospectively analyzed 119 patients treated with ICIs; of them, 24 (20%) had SGLT2i on board. SGLT2i emerged as an independent predictor for lower all-cause mortality [108]. Noteworthy, another important finding was that the baseline hematocrit (HCT) was higher in the SGLT2i group (37 ± 6% vs. 33 ± 6%, *p* = 0.003) underling the active role on hematopoiesis played by these medications in cancer patients [38]. The complex mechanisms through which SGLT2i may oppose ICIs cardiotoxic effects are still unknown and translational research is needed.

**Table 1:** Main immune checkpoint inhibitors (ICIs) available in clinical practice and associated adverse events, Refs. [88–95]

Drug name	Source	Target	Indications	Immune-related adverse events (IR-AEs)	Cardiac immune-related adverse events (IR-AEs) Total (%)^a^	Management of of ICIs-related cardiotoxicity
Pembrolizumab	IgG4 humanized antibody	Programmed cell death protein 1 (PD-1)	Melanoma, lung cancer, head and neck cancer, Hodgkin lymphoma, stomach cancer, cervical cancer	Cutaneous, immune pneumonia, hypothyroidism, joint and muscle pain, colitis, Hepatotoxicity Immune nephritis and pituitary inflammation	Total: 32.5% Myocarditis: 16.3% Pericardial effusion: 8.3% Acute myo- cardial infarction: 1.7% Arrhythmia: 2.4%	1. Withhold ICI therapy (grade ≥ 2) 2. Starting Conventional cardiac treatment 3. Immunosuppressive therapy (if myocarditis or pericardial disease: First line: High-dose corticosteroids (1–2 mg prednisone/kg/day, oral or IV depending on symptoms Methylprednisolone 1 g every day (fulminant myocarditis) Glucocorticoid-refractory and glucocorticoid-resistant: Addition of either mycophenolate, infliximab, rituximab or anti-thymocyte globulin, abatacept (CTLA-4 agonist) or alemtuzumab (CD52 blockade), tocilizumab Plasmapheresis Pericarditis: consider addition of colchicine and nonsteroidal anti-inflammatory drugs
Ipilimumab	IgG1 human antibody	Cytotoxic T-Lymphocyte Antigen 4 (CTLA-4)	Melanoma, renal cell carcinoma (RCC), colorectal cancer, hepatocellular carcinoma, non-small cell lung cancer (NSCLC), malignant pleural mesothelioma, esophageal cancer	Colitis, pituitary inflammation and rash Neurotoxicity (meningitis), hepatotoxicity, Hepatotoxicity, Haematological toxicity, and ocular toxicity	Total: 5.8% (single agent) 9.4% (nivolumab combination) Myocarditis: 9.3% Pericardial effusion: 8.0% Acute myocardial infarction: 4.9 Arrhythmia: 1.2	
Nivolumab	IgG4 human antibody	Programmed cell death protein 1 (PD-1)	Melanoma, lung cancer, malignant pleural mesothelioma, renal cell carcinoma, Hodgkin lymphoma, head and neck cancer, urothelial carcinoma, colon cancer, esophageal squamous cell carcinoma, liver cancer, gastric cancer, and esophageal or gastroesophageal junction cancer	Cutaneous, immune pneumonia, hypothyroidism, joint and muscle pain, colitis, Hepatotoxicity Immune nephritis and pituitary inflammation	Total: 43.2% Myocarditis: 16.4 Pericardial effusion: 6.2 Acute myocardial infarction: 2.4 Arrhythmia: 3.0	
Atezolizumab	IgG1 humanized antibody	Programmed cell death-ligand 1 (PD-L1)	Urothelial carcinoma (UC), non-small cell lung cancer (NSCLC), small cell lung cancer (SCLC), hepatocellular carcinoma and alveolar soft part sarcoma	Cutaneous, immune pneumonia, hypothyroidism, joint and muscle pain, colitis, Hepatotoxicity Immune nephritis and pituitary inflammation	Total: 4.2% Myocarditis: 18.1 Pericardial effusion: 7.9 Acute myo-cardial infarction: 3.9 Arrhythmia: 2.4	

^a^Total Percentages referring to individual case safety reports with at least one ICI as the suspected drug (*n*: 2478) retrieved from Eudravigilance. Date from Mascolo et al. [86]

All these findings identify SGLT2i as potential cardioprotective agents against a wide spectrum of antitumor drugs. Needless to say, further preclinical and clinical studies are much needed to support this hypothesis. However, the translation of data emerged from preclinical studies to clinical practice is limited by the lack of models able to mimic the clinical scenario of cancer patients, often affected by concomitant comorbidities and cardiovascular disease. Indeed, in most studies in vivo models were represented by healthy animals, complicating the transposition of results to elderly, comorbid cancer patients [84, 122]. While assessing the effectiveness of cardioprotective strategies, it is essential to consider the impact of cardiovascular risk factors, such as diabetes or arterial hypertension, and previous cardiovascular events, i.e. acute and chronic coronary syndromes, which frequently affect patients with active cancer or cancer survivors years after drug exposure [171]. Appropriate in vivo models able to integrate the complexity of cancer patients are needed in order to better understand the pathogenesis of cardiovascular toxicity, the relationship between fn cancer and cardiovascular diseases and potential therapeutic options [7, 18].

## SGLT2 inhibitors in cardio-oncology: clinical experience

The promising results of preclinical studies paved the way to the evaluation of potential cardioprotective effects of SGLT2i in patients undergoing anthracycline-based chemotherapy (Table 2). A retrospective study recently included 3033 patients with diabetes mellitus and treated with anthracyclines for solid and hematologic malignancies. After matching subjects for age, sex, and anthracycline starting date, the authors could identify a case population of 32 patients who received SGLT2i during chemotherapy and a control population of 96 subjects who did not. The primary outcome was a composite of HF incidence, HF hospitalization, clinically significant arrhythmias, or a > 10% absolute decline in LVEF to a final value < 53%. During a median follow-up period of 1.5 years, when compared to controls, case patients experienced a lower incidence of the composite primary outcome, as well as reduced overall mortality and incidence of a composite of sepsis and neutropenic fever (16% vs 40%; *p* = 0.013). A non-significant higher incidence of genital infections was observed. The lower incidence of cardiac events was principally driven by a reduction in HF hospitalizations and > 10% decline LVEF to < 53% during chemotherapy [46].

**Table 2:** Available clinical studies exploring the potential cardioprotective role of SGLT2 inhibitors in oncological patients undergoing cardiotoxic treatments

Authors	Population	Pharmacological treatment	Primary endpoint	Results
Gongora et al. 2022 [83]	3,033 patients with diabetes mellitus and cancer (37% of hematologic cancer patients)	SGLT-2i against anthracyclines	Composite of cardiac events (HF incidence, HF admissions, new cardiomyopathy^a^, clinically significant arrhythmias). The primary safety outcome was overall mortality	SGLT2i are associated with a lower cardiac event incidence, a lower overall mortality and a lower composite of sepsis and neutropenic fever
Chiang et al. 2023 [84]	8640 patients with diabetes mellitus and cancer (5% of hematologic cancer patients)	SGLT-2i against anthracyclines, TKIs, and radiotherapy	Hospitalization for HF and all-cause mortality	SGLT-2i are associated with a reduction in hospitalization for HF and a higher overall survival
Abdel-Qadir et al. 2023 [85]	933 patients with diabetes mellitus and cancer (28% of hematologic cancer patients)	SGLT-2i against anthracyclines	Hospitalization for HF, incident HF diagnoses (in- or out-of-hospital), and CVD in future hospitalizations	SGLT2i reduce the rate of HF hospitalization, but not the incidence of HF diagnosis, CVD diagnosis or overall mortality
Avula et. al. 2023 [87]	6,988 patients with diabetes mellitus and cancer (63% of hematologic cancer patients)	SGLT-2i against anthracyclines, alkylating agents, antimetabolites, monoclonal antibodies, small-molecule tyrosine kinase inhibitors, and proteasome inhibitors	HF exacerbations and all-cause mortality	SGLT2i reduce the rate of acute HF exacerbation, all-cause mortality, hospitalizations or emergency department visits, atrial fibrillation or flutter, acute kidney injury, and renal replacement therapy

*CVD* cardiovascular diseases, *HF* heart failure, *SGLT2i* sodium–glucose cotransporter-2 inhibitors, *TKIs* tyrosine kinase inhibitors

^a^Defined as > 10% decline in ejection fraction to  < 53%

In the same way, Chiang et al. conducted a retrospective propensity score-matched cohort study, involving adult patients with type 2 diabetes mellitus diagnosed with cancer (95% with solid tumors and 5% with haematologic malignancies). From a total cohort of 8640 patients, 878 patients received an SGLT2i [primarily empagliflozin (49%), followed by dapagliflozin (38%) and canagliflozin (14%)] while 7556 did not and served as controls. During a median follow-up of almost 20 months, SGLT2i patients showed a three-fold lower rate of hospitalizations for incident HF compared to controls (2.92 vs 8.95 per 1000 patient-years, *p* = 0.018). In Cox regression and competing regression analyses, SGLT2i were associated with a 72% reduction in the risk of hospitalization for HF. Overall, SGLT2i was associated with a higher survival (85.3% vs 63.0% at 2 years, *p* < 0.001), with the risk of serious adverse events (i.e. acute kidney injury and diabetic ketoacidosis) similar in the two groups [24]. Of interest, the use of SGLT2i was associated with a decreased risk of urosepsis, sepsis and hypoglycemia.

Other investigators evaluated a cohort of 933 patients without HF history who were receiving medications for diabetes and underwent anthracycline-based chemotherapy. Of these patients, 99 received an SGLT2i while 834 did not and served as controls. During a median follow-up of 1.6 years, in the SGLT2i population there was a significant lower incidence of HF hospitalizations, while no significant difference in incident HF diagnosis, cardiovascular disease diagnosis or mortality could be detected [1]. Finally, Avula et al. conducted a retrospective investigation on the role of SGLT2i in diabetic patients exposed to antineoplastic agents and developing a decline in left ventricular function. In a cohort of 1280 adult patients without a history of ischemic heart disease but with chemotherapy-induced cardiac dysfunction, patients treated with SGLT2i on top of therapy showed reductions in acute HF exacerbation, all-cause mortality, all-cause hospitalizations, atrial fibrillation/flutter, acute kidney injury and need for renal replacement therapy compared to controls who did not receive SGLT2i [11].

Even if limited by observational design and unmeasured confounders between the populations examined, these studies formed the rationale for randomized controlled trials. The EMPACT (Empagliflozin in the Prevention of Cardiotoxicity in Cancer Patients Undergoing Chemotherapy Based on Anthracyclines; NCT05271162) trial is a multicenter phase III study aiming to assess whether prophylactic empagliflozin may prevent LV dysfunction in patients receiving high-dose anthracycline therapy.

Cardiotoxicity may not only manifest as cardiac dysfunction, but also with arrhythmic events, such as atrial fibrillation (AF) and ventricular tachycardia, generally developing acutely after anticancer treatment administration. In particular, AF is frequent in oncological patients, with an estimated prevalence of 9.77% and an age-related relative risk ratio in patients with cancer compared with no cancer of 10.45 [12]. Pathogenetic factors coming at play are numerous, even though generally involving patients’ related factors, anti-neoplastic therapy performed and/or direct compression/invasion exerted by cancer itself [23, 34, 48, 92, 112]. There is growing evidence about the potential protective role of SGLT2i in arrhythmias and AF [77]. Potential biological mechanisms implicated in antiarrhythmic properties probably involve regulation of Ca^2+^ and Na^+^ homeostasis, as well as the anti-fibrotic and anti-inflammatory effects exerted on myocardial cells [43]. In clinical practice, dapagliflozin showed to influence AF or atrial flutter events in the DECLARE-TIMI 58 population, reducing their occurrence by 19%; this effect was consistent regardless of the patient’s previous history of AF, atherosclerotic disease, blood pressure or history of HF [163]. Likewise, Abu-Qaoud et al. studied the effects of SGLT2i in patients with type 2 diabetes previously diagnosed with AF and treated with catheter ablation, demonstrating that the use of these drugs was associated with a lower risk of arrhythmia recurrence and consequently a reduced need for cardioversion, antiarrhythmic therapy or new AF ablation [3]. In the light of this new evidence, SGLT2i may become an important tool in the management of rhythm-related disorders in the oncological population, specifically in patients treated with cancer therapies associated with arrhythmic events.

## SGLT2 inhibitors and bone marrow function: implications for cancer patients

In addition to the aforementioned cardioprotective effects, SGLT2i seem to exert unique and complex effects on hematopoiesis and bone marrow (BM) function; accordingly, BM cells represent the extrarenal compartment with the highest level of expression of SGLT2, followed by skeletal muscle and myocardial cells [5].

### SGLT2 inhibitors and modulation of erythropoiesis.

The most evident BM effect of SGLT2i is modulation of erythropoiesis. SGLT2i increased hemoglobin count and hematocrit in various randomized controlled trials (summarized in Table 3 along with the suggested mechanisms of action of SGLT2i). The most notable trial to provide such information was the EMPA-REG OUTCOME trial, which recruited 7028 diabetic patients and reported higher hematocrit values in the empagliflozin groups than in the placebo group [170]. While originally ascribed to natriuresis and plasma volume contraction, the increase in hematocrit was later related to an enhanced production of erythropoietin [30, 37, 88]. In fact, an increase in erythropoietin occurred early upon treatment initiation and subsided a new set point for the equilibrium between erythropoietin and haemoglobin levels [153]. SGLT2i improve erythropoietin expression/secretion via at least three distinct mechanisms: modulation of tubulointerstitial hypoxia and hypoxia-induced factor (HIF)‐α expression, mitigation of inflammation-induced functional iron deficiency, and metabolic reprogramming (the latter manifested at a cellular level by an increased production of ketone bodies and a state of starvation mimicry) [104].

**Table 3:** Proposed effects of SGLT2 inhibitors on erythropoiesis

Study	Population	Pharmacological treatment	Haematological effects	Proposed biological mechanism
Zinman et al. 2015 [13]	7020 patients with Diabetes and high cardiovascular risk	Empaglifozin 10 mg–25 mg versus placebo	Increase in Hb and Hct	Increased EPO production via hypoxia-induced activation of HIF2α, modulation of iron metabolism through hepcidin suppression, and hemoconcentration
Maruyama et al. 2019 [124]	9 patients with type 2 diabetes and anaemia of chronic kidney disease	Canagliflozin 100 mg once a day for 12 weeks	Increase in EPO concentration. Increase in Hb and Hct	Improvement of renal tubular interstitial hypoxia, restoring EPO-producing phenotype of interstitial fibroblasts
Mazer et al. 2020 [90]	97 diabetes patients ≥ 40 and ≤ 80 years old	Empagliflozin 10 mg versus placebo	EPO levels significantly increased after 1 month of treatment compared to placebo. Significantly higher Hct, higher Hb concentration and lower mean ferritin levels	Increased oxygen consumption and subsequent tissue hypoxia in the nephron S3 segment, enhancing EPO production Improved oxygenation in the outer renal cortex, restoring dysfunctional interstitial fibroblasts to functional EPO producing cells
Ghanim et al. 2020 [97]	52 obese type 2 diabetes patients	Dapagliflozin 10 mg 12 weeks versus placebo	2-point increase in Hct, significant increase in RBC counts and EPO levels compared to baseline	Increased renal EPO secretion Increased erythroferrone secretion Hepcidin secretion suppression
Thiele et al. 2021 [125]	44 patients with type 2 diabetes	Empaglifozin 10 mg versus placebo	Hct and Hb increase after 3 months of treatment	Reduction of renal cellular stress leading to increased renal erythropoietin secretion

*EPO* Erythropoietin, *RBCs* red blood cells, *Hct* haematocrit, *Hb* haemoglobin, *HIF‐α* Hypoxia-inducible factor alpha

The action of SGLT2i to block Na^+^ reabsorption in the proximal renal tubule leads to increased delivery of sodium to more distal portions of the nephron, where counterregulatory mechanisms of Na^+^ absorption are recruited to limit the magnitude of natriuresis. It has been speculated that SGLT2i might in this way increase oxygen consumption and predispose to tissue hypoxia in the S3 segment of the nephron, possibly in close proximity to the specialized interstitial fibroblast-like cells belonging to the deep cortex, which could be stimulated to produce erythropoietin [156]. On the other hand, inhibition of glucose reabsorption would alleviate metabolic demands in the proximal tubules and reduce oxygen consumption, thus improving oxygenation in the outer renal cortex and allowing dysfunctional fibroblasts to revert to a phenotype that is able to synthetizes erythropoietin [124].

In addition to the aforesaid considerations, the disease states that pose an indication to SGLT2i, such as HF and kidney impairment, are associated with the development of a typical anemia of chronic disease. This is caused by an inflammatory state characterized by increased levels of two major iron regulatory proteins, hepcidin and ferritin, and by a state of functional iron deficiency [147]. Hepcidin, produced by the liver, blocks the absorption of iron from the duodenum and its release from the reticuloendothelial system. SGLT2i reduced serum hepcidin and ferritin levels in both type 2 diabetes [45] and chronic HF [24, 104], in part by stimulating erythropoiesis and in part by a direct anti-inflammatory effect [103]. Regardless of the ultimate mechanism, decreases in hepcidin and ferritin would lead to an increased release of iron from macrophages and intracellular storages, respectively, fueling red blood cells production. A key role seems to be played also by ketone bodies. Acetoacetic acid, β‐hydroxybutyrate, and acetone are in fact hyperproduced in patients during chronic SGLT2i therapy and experimental evidence suggests that β‐hydroxybutyrate infusion causes a concomitant 30% increase in erythropoietin levels and bone marrow glucose uptake in healthy volunteers [71]. If and how precisely an SGLTi-driven upregulation of ketone bodies would contribute to increasing erythropoiesis will nonetheless require further investigations. Finally, SGLT2i up-regulate sirtuin (SIRT)1 [132], which can then activate HIF-2α directly [28] or through heme oxygenase-1 and can enhance transcription of the erythropoietin gene [139]. Thus, SGLT2i may well augment erythropoietin production in kidney and liver cells also in an oxygen-independent manner.

SGLT2i has also been linked with enhanced development and BM release of pro-angiogenic progenitor cells. In particular, dapagliflozin increased BM resident mature leukocytes and circulating white blood cells in both diabetic and non-diabetic mice, and improved diabetes-associated defects in hematopoietic stem cell mobilization by granulocyte colony-stimulating factor, eventually promoting the migration of BM cells to the sites of vascular injury [5]. In humans, empagliflozin increased circulating pro-angiogenic CD133^+^ progenitor cells and increased M2 polarized monocytes with anti-inflammatory properties [51]. As anthracycline cardiomyopathy is at least in part due to impaired mobilization and regenerative functions of endothelial cell progenitors [57], SGLT2i could be of potential value to restore the recruitment of endothelial cell progenitors to sites of injury. As a last observation, the aforementioned work by Gongora et al. [46] showed that SGLT2i reduced a composite outcome of sepsis and neutropenic fever, possibly suggesting that these drugs may also exert some effects on the defense mechanisms of cancer patients treated with chemotherapy. SGLT2i effects on lymphocyte and granulocyte functions could be an interesting field for future research.

### Implications of SGLT2 inhibitors use in clinical practice.

Anemia is a frequent finding in patients diagnosed with solid cancers and lymphomas or acute leukemias, and is further worsened by chemotherapy treatments. Unsurprisingly, anemia is an important adverse prognostic factor for outcomes in lymphoma patients, worsening the overall and progression free survival independent of bone marrow involvement [98]. The pathogenesis of cancer-induced anaemia (CIA) is complex and multifactorial [2]. Cancer-associated chronic inflammatory is an important contributor, leading to a condition of functional iron deficiency; for example, patients with diffuse large B cells lymphoma are characterized by increased circulating levels of IL-6, hepcidin and ferritin, along with a defective erythropoietin production [136]. Current management of CIA relies on iron replacement therapy, transfusions and erythropoiesis stimulating agents (ESA) [16]. However, CIA correction remains an unmet clinical need, considering that only 1/3 of patients respond to ESA. Moreover, ESA is associated with an increased risk of thromboembolic events and concerns around an accelerated tumor progression have been raised. Transfusions of red blood cells, although in some cases unavoidable, seem to worsen prognosis [2]. In this setting, the multifaceted activities of SGLT2i on reducing inflammation while also safely upregulating erythropoiesis hold promise for a better management of CIA. Nevertheless, it is worth noting that SGLT2i-induced erythrocytosis seems not to be accompanied by an increased risk of thrombosis, which makes premature SGLT2i discontinuation or haematocrit lowering strategies (such as implementation of phlebotomy program) unnecessary precautions [41, 42].

**Fig. 4:**
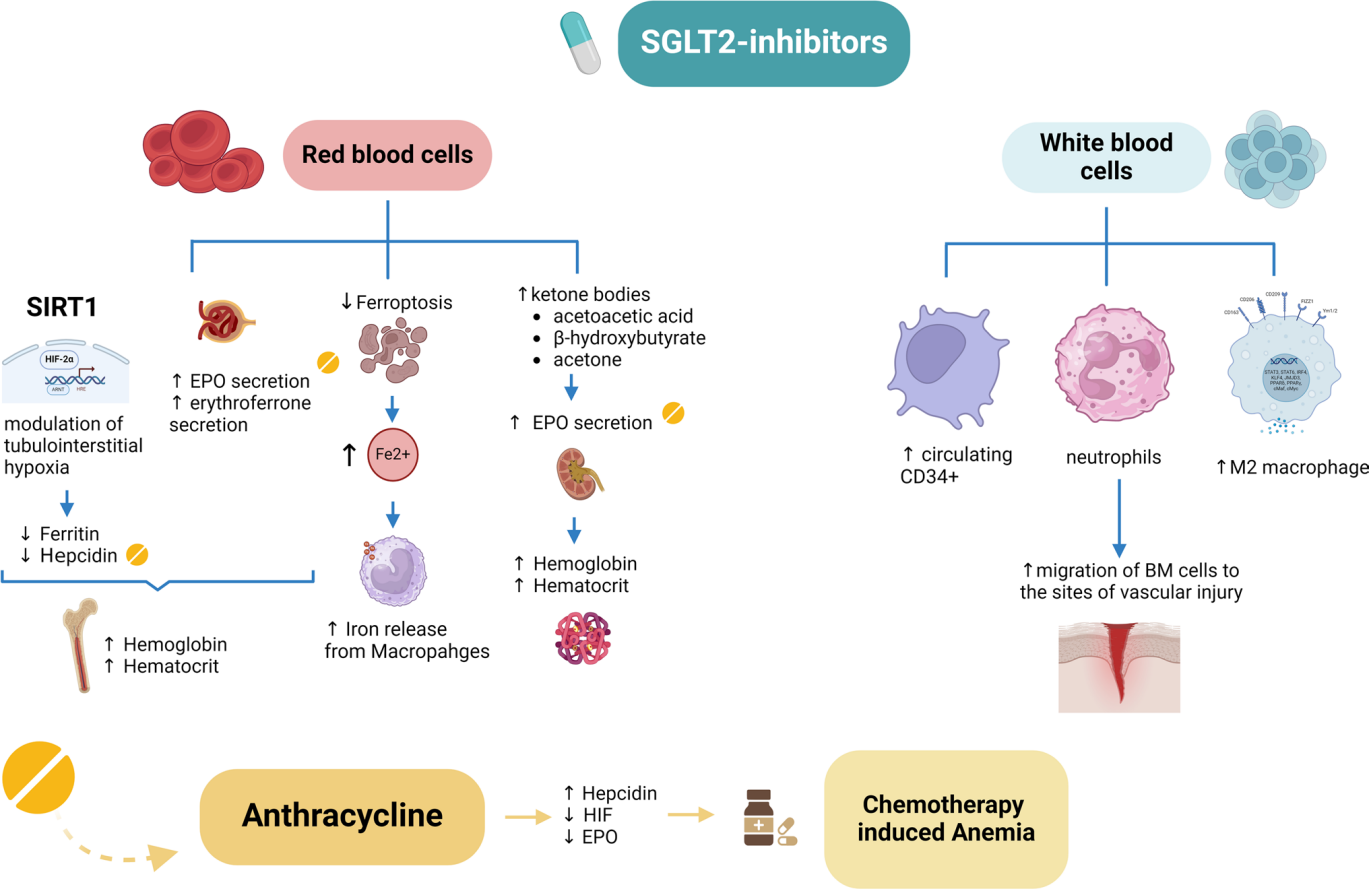
The hematological effects of SGLT2 inhibitors. On the right, the effects on the red blood cell in the context of chemotherapy-induced anemia (CIA): in particular, inhibition of SGL2 promotes the increase in hematocrit by stimulating the release of erythropoietin and activating SIRT1; it induces the modulation of interstitial tubule hypoxia by promoting the reduction of ferritin and hepcidin. SGLT2 inhibitors also reduce Ferroptosis favoring a greater amount of circulating iron from macrophages. On white blood cells, in the mouse models, SGLT2 inhibitors promote the increase of CD34+ cells in peripheral blood and the migration of neutrophils at the level of vascular injuries. Furthermore, they favor the polarization of macrophages into the M2 phenotype. The presence of SGLT2 at the level of lymphoblasts and myeloblasts has not yet been fully elucidated. *EPO* Erythropoietin, *HIF* hypoxia induced factor, *IL6* interleukin 6, *SIRT1* Sirtuin-1

Figure 4 summarizes the role of SGLT2i in the hematologic patient.

## Proposed SGLT2 inhibitors effects on solid tumors and hematological malignancies

There are several in vitro studies suggesting that SGLT2i exhibit anti-proliferative activity against some types of solid tumors, including breast, lung, prostate, colon and pancreas. This may help to design strategies that combine SGLT2i with other cancer drugs [60, 69, 70, 169].

The metabolic reprogramming of cancer cells involves changes in the uptake of various substrates and changes in their metabolic pathways. The most striking example of altered metabolism is the Warburg Effect [78], which consists of increased glucose uptake and its conversion to lactate via anaerobic glycolysis. One of the proposed mechanisms through which SGLT2i may limit tumor growth therefore consists in decreasing glucose availability to cancer cells through inhibition of sodium glucose cotransporter, which in fact is overexpressed in pancreatic, lung and prostate adenocarcinomas and in high grade glioblastomas [127, 148].

In this context, emerging anticancer effect of SGLT2i may also be obtained by reducing the activity of Hexokinase II (HK II), an enzyme that has been found to play a significant role in cancer development. It is overexpressed in many types of cancer cells and has been shown to promote tumor growth and survival by increasing the rate of glucose uptake and metabolism. This enzyme is known to interact with mitochondria, serving as facilitator and gatekeeper of malignancy [87]. HK II is also able to suppress the death of cancer cells, increasing their metastatic potential. For these reasons, targeting this key enzyme is currently being investigated as novel cancer therapies [40]. Recent studies have shown that SGLT2i may have anticancer effects by reducing the activity of HK II. As well, by reducing glucose uptake and metabolism in cancer cells, SGLT2i may attenuate tumor growth and improve the effectiveness of cancer treatments [29, 142, 152]. While both Hexokinase II and SGLT2i have been extensively studied in the context of cancer, further research is needed to fully understand their complex roles in the disease and to develop effective treatments that target these pathways.

Programmed cell death-ligand 1 (PD-L1) function on cancer cells appears to be critical in immune escape and tumor development. SGLT2i promotes PD-L1 recycling, allowing the ubiquitination and proteasome-mediated degradation [27]. As well, a number of emerging evidences suggests that SGLT2i may have direct anticancer effects, going beyond metabolic properties. A recent study demonstrated that SGLT2 is overexpressed in osteosarcoma cells and its inhibition can promote treatment efficacy by inducing T cell infiltration [149]. In the same direction, other recent reports found similar efficacy of SGLT2i in the context of liver [50] and cervical carcinoma [151].

Effects of SGLT2i in solid tumors are currently investigated in small clinical trials. The first trial enrolled 15 participants to assess the tolerability and efficacy of dapagliflozin on top of standard chemotherapy (Gemcitabine and nab-Paclitaxel) in patients with metastatic pancreatic cancer. The drug was well tolerated with favorable changes in tumor response and plasma biomarkers [105]. An ongoing phase 1b/2 study (NCT04073680) plans to recruit 60 participants with advanced solid tumors (breast, endometrial, lung, colorectal, and head and neck cancers) treated in combination with serabelisib (a PI3K inhibitor) and canagliflozin. The study rationale is that hampering the glucose/insulin feedback pathway would enhance the efficacy of PI3K inhibition. A phase I trial (NCT04887935) involving 24 participants is planning to assess the tolerability/efficacy of neoadjuvant SGLT2i before radical prostatectomy in high-risk localized prostate cancer. Finally, a phase 1 study (NCT05521984) will probe dapagliflozin in combination with carmustine for treatment of pediatric brain tumors.

Metastasis is a significant challenge in cancer treatment, often resulting in poor prognosis. Recent research has suggested that SGLT2i may play a role in the prevention of metastasis through different mechanisms: reducing the expression of certain molecules that are involved in the adhesion and migration of cancer cells and reducing the activity of signaling pathways involved in the growth and survival of cancer cells. On this line, a recent study from Rogava and colleagues explored new insights into the mechanisms behind liver metastasis in cancer [118]. In particular, they focused on Pip4k2c (phosphatidylinositol-5-phosphate 4-kinase), an actionable target of SGLT2i, and found that the loss of Pip4k2c leads to the activation of the PI3K-AKT pathway, which plays a crucial role in liver metastasis.

Less is known about SGLT2i as therapeutic agents for hematologic malignancies. A meta-analysis of diabetic patients treated SGLT2i or Dipeptidyl Peptidase (DPP)IV inhibitors found a lower risk of hematological malignancies in the SGLT2i arm, which suggests a possible protective role of these agents [119]. In preclinical studies, SGLT2 was shown to be expressed in the human tonsil [22], in regulatory T cells, memory CD4 and CD8 T cells and NK cells [140]. Moreover, SGLT2 was remarkably expressed in leukemic cells from patients with adult T-cell leukemia (ATL) and SGLT2i suppressed the proliferation of two ATL cell lines and of leukemic cells in peripheral blood from patients with ATL. The antiproliferative effect of SGLT2i in these cells was due to reduced glucose uptake and consequent reduced intracellular levels of ATP and NADPH [99].

On the basis of these preclinical studies, more evidence of SGLT2i in the treatment of hematological malignancies, including the broad spectrum of lymphoproliferative disorders, is highly warranted.

## Conclusions and future directions

We reviewed the promise that SGLT2i hold as cardiac protectants, potential antiproliferative agents and stimulants of erythropoiesis and immune defense in the cancer patient. Overall, this represents the conceptual pillar to foster clinical research and use of SGLT2i beyond currently recommended indications in HF and diabetes. The need for cardioprotective agents is in fact a notable challenge in contemporary Cardio-Oncology, particularly in view of the avalanche of new drugs and therapies heavily treated patients with relapsed or refractory cancer might be considered candidates for. At the same time, the need for improving quality of life and patient reported outcomes in oncologic settings encourage studies of SGLT2i in cancer pathophysiology, immune system regulation and infection control.

## Supplementary Information

Below is the link to the electronic supplementary material.

Supplementary file1 (DOCX 13 KB)

## Abbreviations

AMPK: Adenosine monophosphate-activated protein kinase
AF: Atrial fibrillation
AMPK-mTOR: Adenosine Monophosphate-activated Protein Kinase-Mammalian Target of Rapamycin
ARNI: Angiotensin II receptor blockers-neprilysin inhibitors
ATL: Adult T-cell leukemia
β-OHB: Beta hydroxybutyrate
Ca2+: Calcium ion
CIA: Cancer-induced anaemia
CFZ: Carfilzomib
CKD: Chronic kidney disease
ENaC: Epithelial sodium channel
EPO: Erythropoietin
ESC: European society of cardiology
HR: Hazard ratio
HF: Heart failure
HFmrEF: Heart failure with mildly reduced ejection fraction
HFpEF: Heart failure with preserved ejection fraction
HFrEF: Heart failure with reduced ejection fraction
HNF4: Hepatocyte nuclear factor 4
hs-CRP: Hypersensitive-C-reactive-protein
HIF‐α: Hypoxia-inducible factor alpha
HER2: Human Epidermal Growth Factor Receptor 2
HK II: Hexokinase II
IFN: Interferon
Fe2+: Iron ion
ICIs: Immune checkpoint inhibitors
IL: Interleukin
mmHg: Millimeters of mercury
MyD88: Myeloid differentiation primary response 88
NADPH: Nicotinamide Adenine Dinucleotide Phosphate
NLRP3: NOD-like receptor pyrin domain containing protein 3
PGC-1: Peroxisome proliferator-activated receptor-gamma coactivator
PI3K: Phosphoinositide 3-kinase
Pip4k2c: Phosphatidylinositol-5-phosphate 4-kinase
PD-L1: Programmed cell death-ligand 1
ROS: Reactive oxygen species
RBC: Red blood cells
SGK1: Serum- and Glucocorticoid-Regulated Kinase 1
STAT3: Signal transducer and activator of transcription 3
SIRT1: Sirtuin 1
Na2+: Sodium ion
SGLT2: Sodium–glucose cotransporter 2
TNF: Tumor necrosis factor
TLR: Toll-like receptor
TGF: Transforming growth factor
