# Supplementary material for: Sodium–glucose cotransporter 2 inhibitors and the cancer patient: from diabetes to cardioprotection and beyond
Source: Basic Res Cardiol. 2024 Jun 27;120(1):241–62. doi: 10.1007/s00395-024-01059-9 (PMC11790819; doi:10.1007/s00395-024-01059-9)
Supplement: Supplementary file 1 — Supplementary file1 (DOCX 13 KB) [file 395_2024_1059_MOESM1_ESM.docx]

**Search strategy methodology:**

Regarding the eligibility criteria, the authors searched for studies that examined the use of sodium-glucose cotransporter 2 inhibitors in cancer patients, focusing on their effects on cardioprotection in cancer patients. The authors also looked for studies that met their criteria for study design, including randomized controlled trials, cohort studies, case-control studies, and cross-sectional studies. The study characteristics that were examined included the number of participants, the duration of the study, the dosage of the sodium-glucose cotransporter 2 inhibitor used, and the primary outcome measures. The language of the studies was not restricted, but the authors did note that they only included studies that were published in English.

In terms of publication status, the authors included just published studies available in electronic databases. The scientific field of the journal was also taken into consideration, as the authors only included studies that were published in journals that were relevant to the topic of the review.

Finally, the authors searched electronic databases, including MEDLINE, EMBASE, and Cochrane Central Register of Controlled Trials, to identify relevant studies for the review.
